# The social brain of language: grounding second language learning in social interaction

**DOI:** 10.1038/s41539-020-0068-7

**Published:** 2020-06-19

**Authors:** Ping Li, Hyeonjeong Jeong

**Affiliations:** 1 Department of Chinese and Bilingual Studies, Faculty of Humanities, The Hong Kong Polytechnic University, Kowloon, Hong Kong China; 2 Graduate School of International Cultural Studies & Department of Human Brain Science, Institute of Development, Aging, and Cancer, Tohoku University, Aoba-ku, Sendai Japan

**Keywords:** Human behaviour, Psychology

## Abstract

For centuries, adults may have relied on pedagogies that promote rote memory for the learning of foreign languages through word associations and grammar rules. This contrasts sharply with child language learning which unfolds in socially interactive contexts. In this paper, we advocate an approach to study the social brain of language by grounding second language learning in social interaction. Evidence has accumulated from research in child language, education, and cognitive science pointing to the efficacy and significance of social learning. Work from several recent L2 studies also suggests positive brain changes along with enhanced behavioral outcomes as a result of social learning. Here we provide a blueprint for the brain network underlying social L2 learning, enabling the integration of neurocognitive bases with social cognition of second language while combining theories of language and memory with practical implications for the learning and teaching of a new language in adulthood.

The study of the neuroscience of cognition has made great strides in the last two decades, thanks to the rapid developments in non-invasive neuroimaging techniques and the corresponding data analytics. At the same time, the study of language acquisition, including second language (L2) learning by children and adults, has also progressed significantly from behavioral research toward neurocognitive understanding, thanks also to new methods including neuroimaging. These two domains of study (i.e., cognitive neuroscience and language learning) have seen increasingly happy marriages of approaches, theories, and methodologies in the last two decades, driven largely by the *New Science of Learning*^1^, a framework for studying learning at the intersection of psychology, neuroscience, education, and machine learning. Specifically, this framework argues that learning should be studied along three important dimensions: a computational process, a social process, and a process supported by brain circuits linking perception and action. Meltzoff and colleagues^1^ further suggested that human language acquisition provides a *bona fide* example for connecting computational learning, social learning, and brain circuits for perception and action. Despite the call from this multi-disciplinary perspective, researchers in cognitive neuroscience and language acquisition have remained to focus on the individual learner, especially in the study of adult L2 learning. This tradition has seriously limited our understanding of a key aspect of what it means to learn: learning in the social context, interactively.

The study of language learning focused on the individual might have had its origin in the tradition of generative linguistics^2,3^, according to which linguistics as a science should study the language competence of the idealized speaker and the corresponding innate mechanisms that enable humans to learn language. Although the neuroscience of language has largely avoided accepting the generative tradition, the focus on the individual, and consequently, the brain structure and function of the individual (i.e., the “single-brain” approach^4^), has not changed as a field (see Fig. 1 for illustration*)*. This is unfortunate, since language serves a social communicative purpose and is fundamentally a social behavior. Note that there are some exceptions to this focus, especially in (a) the study of child language learning (see discussion next), and (b) social neuroscience, which has begun to focus on how brains respond to social interactions using methodologies such as hyper-scanning^4,5^. In addition, although leading models of the neurobiology of language do not incorporate a social component^6^, there have been recent efforts to extend the landscape to include pragmatic reasoning^7^, theory of mind^8^, and social interaction^9^.

**Fig. 1:**
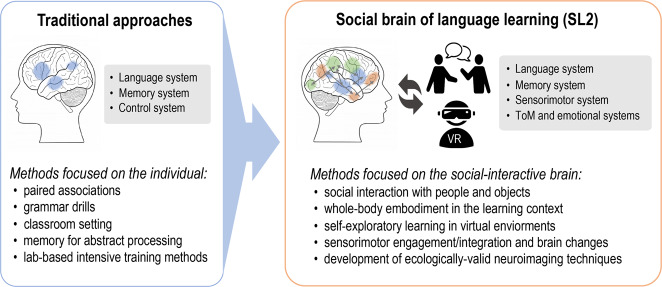
Contrasting frameworks and methods. Left: Traditional approaches for “single-brain” study of language learning; Right: “Social-interactive brain” research and emerging methods.

In this paper, we advocate an approach focused on grounding L2 learning in social interaction; we call this approach “Social L2 Learning” (SL2). Specifically, we define “social interaction” here as “learning through real-life or simulated real-life environments where learners can interact with objects and people, perform actions, receive, use, and integrate perceptual, visuospatial, and other sensorimotor information, which enables learning and communication to become embodied.” Notwithstanding generative linguistics and individual-brain study approaches, the field of first language (L1) acquisition has clearly demonstrated that children, from the earliest stages, depend on social interactions to learn^1^. This dependence may be initially coordinated through “joint attention” and shared intentionality between the infant and the parent/caregiver^10^. Computational models that incorporate social-interactive cues from mother–child interactions perform significantly better than models with no such cues included^11,12^. Kuhl et al.^13^ further indicated that social learning is crucial even when children learn an L2: American babies exposed to Mandarin Chinese through a “DVD condition” (pre-recorded audiovisual or audio-only material) did not demonstrate learning of Mandarin phonetic categories as did babies who were exposed to the same material through a “live condition” (experimenter interacting with the infant during learning). For adults, however, folk wisdom suggests that they can learn an L2 rapidly without social cues (e.g., through intensive training in a classroom) and may be less dependent on the presence of peer learners. Limited evidence, however, suggests that social cues such as joint attention may also enhance L2 learning success through orienting the learner’s attention to the correct meaning among competing alternatives^14^.

## Theoretical frameworks for understanding the social brain of language Learning

The proposed SL2 model is focused on grounding L2 learning in social interaction based on both behavioral and brain data. A number of important theoretical framworks have already paved the way for the SL2 model, some of which are separately known in the domains of psycholinguistics, memory, and cognition, respectively.

First, while the classic Critical Period Hypothesis^15^ suggests a biology-based account of effects of age of acquisition (AoA) on learning, the Competition Model, in its various formulations^16–20^, provides a social-based and interactive-emergentist account of the differences between L1 and L2 learning. Upon this account, the principles of learning are not fundamentally different between the child learning an L1 and the adult learning an L2 (e.g., contra the “less is more” hypothesis^21^), but the processes and contexts within which learning takes place may be significantly different. For children, language learning is a natural event that unfolds in the environment where they grow up. They can naturally integrate the rich perceptual and sensorimotor experiences from this environment, interacting with the objects and people and performing actions in it. Picking up and using a spoon while hearing the sound “spoon” is part of the learning process, which differs from the process where adults sitting in the classroom look at a picture of spoon and associate it to an existing label in their native language. According to MacWhinney^17^, adult L2 learning is susceptible to several major “risk factors”, factors that prevent adults from acquiring a foreign language to native competence. These include thinking in L1 only (which implies the need to translate from L2 to L1 rather than directly using L2 as a medium), social isolation (learning as an individual or through in-group communities only), and lack of perception-action resonance (lack of direct contact with the target objects or actions in the environment while learning L2). These risk factors, particularly social isolation and lack of perception-action-based contexts, may explain why adult learners display the strong parasitic L2-on-L1 representations^22^: on the one hand, adults typically start to learn L2 when they have already established a solid L1 (“entrenchment” in L1), which lends easily to L2-to-L1 translation and association; on the other hand, they lack a dynamic and variable environment to build direct relations between L2 words and the objects/concepts to which the words refer^23^. With regard to the risk factors of thinking in L1 and social isolation, empirical evidence has shown that study-abroad experience may provide some environmental support, particularly in attenuating L1 to L2 interference for late adult learners^24^.

These theoretical perspectives are consistent with a larger trend in psycholinguistics to examine language learning and bilingualism not as an individualized but a general communicative experience. Adults show significant differences in how they learn two (or more) languages, the frequency and contexts with which they use the languages, and the communicative purposes for which each language is needed, therefore showing that bilingualism is a highly dynamic developmental process^19,25–27^. The SL2 approach advocated here also echoes a movement in the broader language science, from sociocultural theory^28^ to usage-based language learning^29^ and conversational analysis^30^, all of which view language learning as a socially grounded process. Ellis^31^ summarizes this movement with regard to its focus on “how language is learned from the participatory experience of processing language during embodied interaction in social and cultural contexts where individually desired outcomes are goals to be achieved by communicating intentions, concepts, and meaning with others.”

Second and independently, human memory research suggests that item-based learning (encoding) and use (retrieval) are highly interdependent. This is due to the associative nature of memory, in which the cognitive operations used for encoding stimulus items directly impact their subsequent retrieval. A well-established hypothesis in this regard is the “encoding-specificity” principle^32^, according to which semantic memories are more successfully retrieved if they are recalled in the same context as when they were originally encoded (e.g., if word lists were encoded underwater they would be recalled better underwater than on dry land^33^). Related to this hypothesis is the “levels of processing” theory^34^ that suggests deeper, more elaborative, or richer semantic processing during encoding would lead to more successful retrieval than shallow or surface-level processing of the same material. If encoding involves more elaborative semantic processing, e.g., using multimodal information, it will have a positive impact on memory retention and retrieval. Both the “dual encoding” theory^35^ and the multimedia learning theories^36^ suggest that elaborative processing with multimodal sensory information could enhance the quality of semantic memory, hence leading to better recall. One of the predictions here is a “multimodal advantage” such that, for example, people learn better with words and pictures together than with words alone^37^.

There have been several studies that build on the encoding-specificity principle to account for bilingual language processing. Marian and Kaushanskaya^38^ proposed a language-dependent memory hypothesis to explain bilingual semantic/conceptual representation, according to which language is encoded in the episodic memory of an event and therefore forms part of one’s autobiographical memory. It is this episodic encoding that influences the accessibility of semantic memories. They observed that memories were more accessible when retrieved in the same language in which they were originally encoded or learned. Furthermore, this language specificity in bilingual memory is influenced by variables such as AoA, proficiency in the L2, and history of usage in the two languages^39,40^; for example, richer memories were associated with an earlier age of L2 learning.

The richness of memory with regard to AoA may be explained by the rich episodic experiences/events associated with specific perceptual-sensory features in the environments, perhaps because early L1 learning includes these experiences but late L2 learning typically does not. This leads us to the embodied cognition theory^41,42^, according to which *body-specific* (e.g., head, hand, foot) and *modality-specific* (e.g., auditory, visual, tactile) experiences form an integral part of the learner’s mental representation of concepts, objects, and actions. This contrasts with classic cognitive theories of symbolic representation that argue that cognition and cognitive operations are modular, and that language is unrelated to the rest of cognition including perception and action^43,44^. The embodied cognition theory highlights the *whole-body interaction* with the context, that is, “interaction between perception, action, the body and the environment”^45^, and when engaged, will also activate the brain’s perceptual and sensorimotor cortex^46,47^. Although the embodied cognition hypotheses have been examined in many studies of brain and behavior, so far, the focus has been on native L1 speakers; whether and how body-specific and modality-specific experiences play the same role in L2 learning has not received much attention^48,49^. Our SL2 model argues for the important role of social interaction for L2 learning and draws on the link between learning and perception and action, as suggested by the *New Science of Learning* framework^1^.

## Social interaction for second language learning: neuroimaging evidence

How do the theoretical frameworks above shed light on our SL2 approach in understanding the social brain of L2 learning? Although many recent neuroimaging studies have examined brain changes resulting from L2 learning^50^, most of this literature has focused on traditional L2 learning methods such as rote memorization or translation-based learning, in either classroom settings or lab-based intensive training^51–55^. Their findings suggest largely the engagement of language-related neural networks (e.g., the classic frontal-parietal network) and memory-related brain regions (e.g., the medial temporal region for the learning and consolidation of linguistic information; see Fig. 1 for illustration). So far, only a handful of studies have provided initial evidence on the neural networks implicated in social-based L2 learning, pointing to the following key patterns.

First, the supramarginal gyrus (SMG) and the angular gyrus (AG) could play a significant role. In one of the first studies in this domain, Jeong et al.^56^ trained Japanese speakers to learn Korean words under two conditions, either through L1 translation or simulated social interaction in which the participants watched videos that showed joint activities in real-life situations (e.g., the L2 target word “Dowajo”, meaning help me in English, is shown in the video with an actor trying to move a heavy bag and asking another actor for help). The authors then asked participants to retrieve the target L2 words in a functional magnetic resonance imaging (fMRI) session. The results indicated that the words learned through videos with social interactions produced more activation in the right SMG whereas the words learned from translation produced more activity in the left middle frontal gyrus (MFG). Interestingly, retrieval of L1 words (acquired by these participants in childhood through daily life) also produced greater activation in the right SMG. These findings can be interpreted to suggest that L2 words learned via social interaction (as simulated in videos through short-term training) are processed in a similar fashion as L1 words.

Second, the right inferior parietal cortex (IPL, including both SMG and AG) has been implicated more strongly in virtual reality-based (VR) interactive learning as compared with non-virtual, word-to-picture association, learning^57^. Legault and colleagues found that cortical thickness, a structural brain measure of gray-matter thickness from the surface of the cortex to the white matter, is associated with different contexts of learning: after 2–3 weeks of intensive L2 vocabulary training across seven sessions, the VR learners showed a positive correlation in the right IPL with performance across all training sessions, while the non-VR learners showed a positive correlation at the final stages only in the right inferior frontal gyrus (IFG), a region associated with effective explicit language training^58^ (though there is counter evidence^59^). Furthermore, cortical thickness in the right SMG was correlated with higher accuracy scores of the delayed retention test, but only for the VR learning group. The VR group was engaged in 3D virtual environments in which the learners could dynamically view or play with the objects in an interactive manner.

Third, the right SMG is shown to be more activated in simulated partner-based learning than individual-based learning of word meanings, indicating that the mere presence of a social partner would facilitate L2 word learning^59^, like in child language learning. Verga and Kotz^59^ further found that participants with higher learning outcomes showed higher activity in the right IFG during an interactive learning condition but not during an individualized non-interactive learning condition. Levels of activity in the right lingual gyrus (LG) and right caudate nucleus (CN), previously implicated in visual search process and visuospatial learning, were also found to correlate with temporal coordination between a learner and a partner during simulated interactive learning.

These brain imaging data suggest that social-based L2 learning versus classroom-based individual learning conditions can lead to distinct neural correlates; for example, social learning of L2 may engage more strongly the brain regions for visual and spatial processing^57,59^, which may have consequences on both encoding (learning) and retrieval of information (memory). In contrast to the idea that only the child brain may respond to social learning, these findings suggest that the adult brain displays significant neuroplasticity in response to social interaction. Jeong et al.^56^ showed that if an L2 word was initially encoded in a more socially interactive condition (through video simulations), it engaged the relevant brain areas as in L1, areas that would not become activated if learning had occurred through word association or translation as in a typical L2 classroom.

**Fig. 2:**
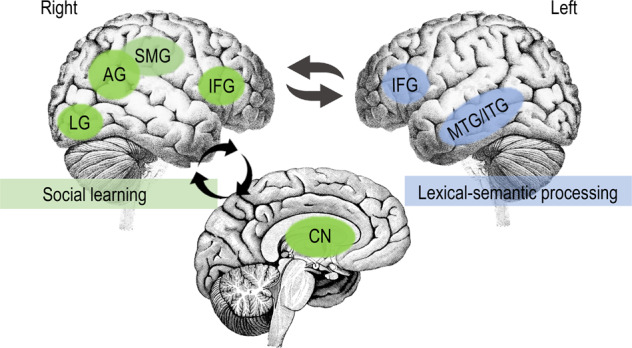
The neural network underlying SL2 that engages the IPL-IFG-CN plus visual cortex loop. The left hemisphere regions (blue) handle lexical-semantic processing, while the right hemisphere cortical plus the subcortical regions (green) participate in social learning. IFG inferior frontal gyrus, SMG supramarginal gyrus, AG angular gyrus, LG lingual gyrus, CN caudate nucleus, MTG/ITG middle temporal gyrus/inferior temporal gyrus (Note: the right hemisphere is depicted on the left side and left hemisphere on the right side).

Figure 2 illustrates the proposed neural correlates of social interaction in the frontal, parietal, and subcortical regions for L2 learning. The strong engagement of the SMG, AG, IFG, along with the visual (LG) and subcortical regions (CN), may form an important neural network for understanding how SL2 is instantiated in the human brain. Importantly, this network highlights the stronger role of the right-hemisphere brain regions as compared with the typical left-lateralized language networks. The IFG has long been implicated in lexical-semantic processing and its integration with memory^60^, which is shown bilaterally in both hemispheres in Fig. 2. The other regions, the SMG, AG, LG, CN, are illustrated in Fig. 2 on the right hemisphere. The role of this “right-heavy” network is evidence of the significant neurocognitive impacts of social L2 learning as opposed to traditional methods (Fig. 1).

There are a number of important issues for further consideration with regard to the SL2 network charted in Fig. 2. First, it is important to understand how the various areas collaborate and communicate with each other during learning and memory. A true brain network is one that involves modules, communities, and pathways that are dynamically connected and organized. An important research direction in neuroscience today is the network science approach towards the analysis of functional/structural brain patterns underlying cognition, and significant advances have been made in applying this approach to the understanding of neural circuits of learning and memory, including L2 learning^61–63^. It remains to be understood how the left frontal IFG and right parietal IPL areas (including SMG and AG) form a dynamic network in support of SL2 learning, alongside the visual and subcortical regions (LG and CN). It is possible that the LG and CN regions play an important early role in visuospatial analysis and learning in social settings, which feeds into action-based lexico-semantic and conceptual integration that heavily involves the SMG and AG regions, as evidenced in studies by Verga and Kotz^59^, Jeong et al.^56^, and Legault et al.^48^. The IFG then coordinates this network with significant participation of semantic memory and cognitive control as well as lexical retrieval^64^. In this regard, the IFG also plays a significant role in modulating competition between L1 and L2 in a language control network^19,65^.

Second, a related issue for further study is how such neural networks evolve during development, which would allow us to understand the degree to which time of learning (e.g., AoA), extent of learning, and increased proficiency may impact the dynamic changes in the neural network^50^. Elsewhere significant progress has been made in this domain^20,66,67^, but the focus there has been on the relationship between cognitive control and bilingualism and the related debate on bilingual cognitive advantage (see a recent discussion^25^). Methodologically, to study the developmental process we will also need to pursue longitudinal neuroimaging work^51^ as well as short-term intensive training paradigms. Finally, much work is needed for understanding how the SL2 network may overlap with neural networks implicated in other types of social interaction^68,69^. Hagoort and Indefrey^7,70^ suggested that pragmatic inference in language processing involves the “theory of mind” (ToM) or the mentalizing network^71,72^, in which the medial prefrontal (mPFC), along with the temporoparietal junction (TPJ) regions, play an important role in social reasoning such as thinking about other people’s beliefs, emotions, and intentions. Not surprisingly, the extended language network (ELN) hypothesis for narrative text comprehension^73^ significantly overlaps with the ToM network, involving mPFC and the TPJ in building story coherence, drawing inference, and interpreting pragmatic meaning in the narrative story being read. The ELN network allows the reader to follow the plots, empathize with the characters, and take the protagonist’s perspectives^74,75^. We hypothesize that the SL2 network in Fig. 2 dynamically connects to mPFC and TPJ implicated in ToM and social reasoning, although this hypothesis needs to be examined carefully by comparing learning with social interaction versus without.

## New approaches toward SL2 as a theoretical hypothesis and a practical model

### Embodied semantic representation in L1 and L2

In a typical adult L2 learning setting, students rely on translation/association of two languages and rote memory, unlike the child who acquires the L1 with sensorimotor experiences in an enriched perceptual environment. For example, in an L2 classroom, the teacher introduces a new L2 word (e.g., Japanese “inu”) by its translation equivalent in the L1 (e.g., English “dog”) and the learner’s task is to form paired associations between L1 and L2 when learning the L2 vocabulary. Although this method is efficient early on, it leads to what is called a *parasitic* lexical representation: the L2 word is conveniently linked to a conceptual system already established through the L1^19,22^. Because the task of word association or translation does not encourage direct L2-to-concept relations, the link from the L2 word to the concept is weak, and has to be indirectly mediated via the L1-to-concept link^23^. More significantly from the SL2 perspective is the “collateral damage” of this parasitism: the new L2 representation lacks the relevant perceptual-spatial-sensorimotor features (e.g., shape, size, motion and location of “inu” or dog), features that are an integral part of the lexical-semantic representation in the L1.

Why can’t the adult L2 learner take the newly acquired L2 representation and map it to the rich embodied features in the L1 representational system, given that would be the most efficient way? Several computational models^22,76,77^ have systematically manipulated the timing of adding new L2 items to L1 lexical structure during simultaneous or sequential learning of the two languages and showed that the L2 lexical organization is sensitive to AoA: the later L2 is learned, the less well organized and more fragmented the L2 representations are. Thus, parasitism is characteristic of L2 semantic learning in late adulthood. Hernandez et al.^19^ and Li^78^ attributed this to the mechanism of “entrenchment”, in which the lexical structure established by the L1 early on is entrenched to resist radical changes during later L2 learning. The entrenchment may have led to late adult L2 learner’s inability to map L2 forms directly to the rich L1 lexico-semantic representations. In a recent neuroimaging study comparing L1 vs. L2 embodied semantic representations, Zhang et al.^79^ showed that L1 speakers engage a more integrated brain network connecting key areas for language and sensorimotor integration during lexico-semantic processing, whereas L2 speakers fail to activate the necessary sensorimotor information, recruiting a less integrated embodied brain system for the same task.

The persistent parasitism could also be attributed to the different contexts in which the two languages have been learned. Recent evidence from affective processing indicates that affective-specific experiences are more strongly evoked in L1 than in L2 words due to the different contexts of social learning (e.g., family vs. workplace interactions) and the co-evolution of emotional regulation systems with early language systems^80–82^. Consistent with embodied semantic differences^79^, such emotionality differences between L1 and L2 have been found most reliable when the L2 is a later-learned or less proficient language^80^, showing evidence that the L2 representation, if acquired late, cannot easily incorporate the rich social and affective features of the L1 representation.

How can the L2 learner break away from this parasitism so as to establish the L2 representation on a par with the L1 representation? SL2 provides a theoretical framework for addressing this question from an embodied cognition perspective. Recent work suggests that embodied actions, even when no direct social interaction is involved, can impact learning outcomes simply by engaging the body, for example, through gestures. Mayer et al.^49^ showed neurocognitive differences between (a) L2 vocabulary learning with gestures that activated the superior temporal sulcus, STS, and the premotor areas, versus (b) learning without gestures that activated the right lateral occipital cortex only. Critically, learners in the gesture condition showed significantly better memory for L2 words, hence more sustained retention, than the non-gesture learners, even after 2–6 months. Such findings point to the significance of embodied “body-specific” (hands in this case) activities for learning, and are consistent with the sensorimotor-based neural accounts of semantic representation^20^. According to the “hub-and-spoke model”^83,84^, “modality-specific” versus “modality-independent” (or “amodal”) representations are realized in different neural circuits, in visual/auditory/motor areas versus anterior temporal lobe, respectively. However, the outcome conceptual system must encode knowledge through integrating higher-order relationships among sensory, motor, affect, and language experiences. In this regard, one of the outstanding questions raised by Pulvermüller^84^ was whether semantic learning from embodied experience and context could lead to different semantic representations in the mind and the brain. This question becomes particularly relevant when we examine the contexts of SL2 learning.

### Simulated social interaction, technology, and the brain

In addition to the cognitive and neuroscience models that support SL2 theoretically, recent advances in technology have enabled us to study SL2 as a practical model toward building embodied representations in the L2 through technology-based learning. Because of the L1 vs. L2 embodied representation differences^79^, the L2 learner should aim at integrating modality-specific information with the newly acquired L2 amodal representations, in order to fully approach native-like conceptual-semantic representations. Technology-based learning could aid in this process from the earliest stages of learning, given the ample evidence from (a) technology-enhanced child language learning^85,86^, (b) prevalence of technology-based multimedia learning for both children and adults^36^, and (c) evidence of multimedia learning effects on the brain^37^. For example, in child language, despite a clear advantage of live learning compared to screen-based DVD learning^13^, it is now shown that direct face-to-face human interaction is not a necessary condition for infant foreign language learning. Children can benefit from technology such as Skype and other screen media platforms, provided that these technologies can deliver simulated social interactions, for example, through video chats^85^. Lytle et al.^86^ showed that when the same learning materials from Kuhl et al.^13^ were delivered to children through play sessions with an interactive touchscreen video, children can indeed learn from the videos. This study clearly points to both the role of interactive social play (simulated through touchscreen videos) and the impact of technology, breaking the simple dichotomy between live human learning (as effective) vs. screen-based learning (as ineffective).

In real-life learning situations, students observe and integrate multiple sources of information including actions and intentions of the speaker for using specific words in specific contexts. In a follow-up study of Jeong et al.^56^, Jeong et al.^87^ examined fMRI evidence during learning (i.e., encoding), under both traditional translation and simulated social interaction conditions. The authors controlled for the amount of visual information in the two conditions by using L1 text and L1 videos as baseline comparisons. In the simulated video condition, participants had to infer the meaning of L2 target words by observing social interactions of others. Learning of L2 words in this condition resulted in additional activation in the bilateral posterior STS and right IPL. Compared with learning through L1 translation, this condition also resulted in significant positive correlations between performance scores at delayed post-test and neural activities in the right TPJ, hippocampus, and motor areas.

Jeong et al.’s new findings showed that simulated social interaction methods, compared with traditional translation/association methods, may result in stronger neural activities in key brain regions implicated for memory, perception and action, which can boost both recall and sustained long-term retention. These results are consistent with the semantic memory encoding and retrieval theories reviewed earlier. They are also consistent with recent multimedia learning effects on the brain, reflected in the bimodal encoding advantage that materials learned in multimodal conditions (e.g., learned from videos that engage both auditory and visual channels^37^) may lead to sustained neural activities in AG, mPFC, hippocampus, posterior cingulate, and subcortical areas. These brain areas, including mPFC, TPJ, and hippocampus, significantly overlapped with the SL2 brain network and the ToM network that relies on social learning and reasoning (Fig. 2).

Videos or other multimedia platforms, although very effective as discussed, nevertheless have their limits with regard to social interaction and “whole-body” embodiment/engagement as in real life. Recent technological advances in immersive technologies (e.g., virtual reality, VR and augmented reality, AR) enable social interaction to a greater extent, by simulating real-world contexts and promoting student learning through active and self-exploratory discovery processes^88^. VR also provides a new platform to connect cognition, language learning, and social interaction, as it allows researchers to simulate the process of learning in its natural ecology without sacrificing experimental rigor^89,90^. In the current consideration, and in light of Competition Model and Embodied Cognition theories discussed, VR provides a tool for students to learn L2 in a new way. Specifically, it enables the adult learner, like the child L1 learner, to directly map (“perceptually ground”) the L2 material during learning onto objects, actions, and episodic memory to form embodied semantic representations in the L2.

Although VR has been applied to L2 teaching and learning, systematic and experimental research is still scarce in understanding the effects of VR as a function of both features of the technology and characteristics of the learner^90^. Lan et al.^91^ and Hsiao et al.^92^ provided early evidence in this regard. The authors trained American students to learn Mandarin Chinese vocabulary through *Second Life*, a popular desktop virtual platform of gaming and social networking, and demonstrated that (a) the virtual learners needed only about half of the number of exposures to gain the same level of performance as learners through traditional associative learning, and (b) virtual learners showed faster acceleration of later-stage learning. More importantly, clear individual differences in learning were observed: the low-achieving learners tended to follow a fixed route in the virtual space (using the “nearest neighbor” strategy to learn), whereas the high-achieving learners were more exploratory, grouping together similarly sounding words or similarly looking objects for learning. Interestingly, such individual difference patterns could be captured by statistical methods such as “roaming entropy” to quantify the degree or variability of movement trajectories in self-directed exploration of space, a measure previously shown to correlate with neural development during spatial navigation^93^: better learners showed higher roaming entropy, indicating more exploratory analyses of the virtual environment. Thus, navigation patterns in the VR may reflect how learners conceptually organize the environment and their abilities to explore it interactively.

**Fig. 3:**
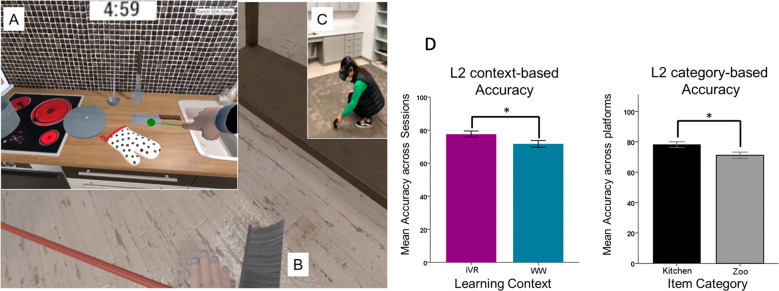
Immersive VR (iVR) learning. **a** In the iVR kitchen, the learner used her handset to point to any item and hear the corresponding word (e.g., “dao”, Chinese knife in the example). **b** The learner could pick up and move objects (broom in the example) by pressing a trigger button with index finger; (**c**) position of the learner picking up the item (broom)—the learner consented to the use of her photo here. **d** Left panel: Effect of learning context (iVR vs. word-word association); Right panel: effect of category of learning (iVR kitchen vs. iVR zoo). Error bars indicate 95% confidence intervals (CIs). * indicates significant effect (from Legault et al.^48^; copyright permission from MDPI).

Most L2 virtual learning studies, like Lan et al.^91^, have relied on desktop virtual platforms like *Second Life* rather than more interactive and immersive VR (iVR). Limited evidence suggests that iVR, with its more realistic simulation of the visuospatial environment and more bodily activity and interaction, leads to higher accuracy in memory recall tasks^94^. It is likely that iVR, compared to desktop VR, more strongly engages the perceptual-motor systems and maximizes the integration of modality-specific experience, and therefore generates better embodied representation^90^. In Legault et al.^48^, participants wore head-mounted displays to view and interact with objects/animals in an iVR kitchen or zoo, and showed significantly better performance of L2 vocabulary attainment than learning through the L2-to-L1 word-to-word association method. Further, the kitchen words were learned better than the animal words, presumably because the learner could more directly manipulate the virtual objects in the kitchen (e.g., squatting and picking up a broom and moving it around; see Fig. 3 for an illustration) than they could with the virtual animals in the zoo. The iVR kitchen environment thus conferred more “whole-body” interactive experience to the learner, especially with respect to the engagement of the sensorimotor system^95^.

In terms of the SL2 framework, VR has the promise of providing a context of learning for children and adults on equal footing, and in particular, it simulates “situated learning”, a condition whereby learning takes place through real-world experiences and visuospatial analyses of the learning environment, experiences and analyses that are often absent in a typical classroom^88^. Therefore, the positive benefits of SL2 learning based on either real or simulated social interactions are clear, including at least the aforementioned aspects of (a) embodied, native-like, neural representation^56^, (b) more sustained long-term memory^49^, and (c) less susceptibility to L1 interference^24^. These benefits not only apply to foreign language learning, but also other educational contents such as spatial learning and memory^90^ and learning of subjects in STEM (i.e., science, technology, engineering, and mathematics)^88^.

VR is an excellent example of the power of today’s technology-based learning, and it urges us to study how students can take advantage of rapidly developing technologies for better learning outcomes. We need to pay attention to the specific key features that support VR learning (e.g., immersive experience, spatial navigation, and user interactivity), the individual differences therein (e.g., cognitive characteristics of the learner including memory and motivation), and the underlying neurocognitive mechanisms (e.g., sensorimotor integration) that enable VR as an effective tool^90^. In this regard, the SL2 approach we advocate here will have the potential of not only benefitting students in terms of reaching native-like linguistic representation and communicative competence, but also providing specific recommendations to teachers in the classroom, especially for those struggling students who may need help in integrating multiple sources of information through contextualized learning. For example, as indicated by Legault et al.^48^, it is the struggling students (“the less successful learners”) who benefitted more from VR learning than from non-VR learning, whereas for the successful learners, VR versus non-VR learning did not make a significant difference. Consistent with the larger trend in education to promote personalized learning and active learning in STEM^96^, there is a movement for today’s classroom instructions to be structured differently from the traditional “teacher-centered” instructional methods, to encourage more “student-centered” interactions and in-depth discussions (e.g., the “flipped classroom” model). E-learning technologies including VR play a significant role in this movement.

### Future directions

New exciting research in the neurocognitive mechanisms of SL2 has just begun. To understand different aspects of L2 learning from a multi-level language systems and multiple networks perspective^7^, neuroimaging studies should extend their focus from the lexico-semantic level to phonological, morphological, syntactic, and discourse levels with the SL2 approach. For example, if, as in infant L1 learning, L2 phonology can be learned through socially enriched linguistic exposure (e.g., multi-talker variability, visible articulation), then even late L2 adult learners may advance to native competence^97^. It is also important to examine how social interaction impacts the acquisition of different types of syntactic rules (e.g., cross-linguistically different syntactic features), as demonstrated in a recent fMRI study of the acquisition of possessive constructions in Japanese Sign Language^98^. The relationship between lexical versus syntactic acquisition is also a topic of significant research interest. While lexical learning typically elicits stronger involvement of the declarative system, morphosyntactic learning likely involves to a greater extent the procedural memory system^99,100^. How L2 lexical learning may also engage the procedural memory system in light of the SL2 brain network (Fig. 2) needs to be seriously considered and carefully examined in future studies.

Despite the significant effects of social L2 learning, individual differences have been observed as discussed^48,92^. It is therefore important to examine in greater detail both the contexts of learning and the characteristics of the learner^90^. Specifically, the magnitude of the effects might depend on the interaction between features of social learning and the learner’s cognitive and linguistic abilities. It is possible that highly interactive, embodied experiences are more helpful to some than to others^48^: learners who are poor at abstract associative learning may benefit more from social-interactive learning. A challenge to future research will be to identify the nature of the interaction between the individual learner’s inherent abilities and the richness of the social learning context.

Finally, a number of new directions present further research opportunities. For example, systematic investigation is needed for understanding the role of various types of non-verbal information that may contribute to positive L2 learning outcomes. Previous cognitive neuroscience studies have provided empirical evidence that non-verbal information (e.g. gesture, communicative intention) facilitates speech comprehension and production, as well as language learning in children and adults^49,101,102^. Furthermore, it is important to study how SL2 facilitates affective processing such as emotion and motivation^81,103^ and consequently how it engages the brain’s limbic and subcortical reward systems. As discussed earlier, there is evidence that emotional responses are more strongly associated with L1 than L2 and social contexts may be a significant contributor to this association^80,81^. Indeed, social interaction has been studied as one of the most crucial contributors to the development of learning motivation in L2 acquisition^104,105^. The SL2 approach provides a framework for integrating previous findings and hypotheses with new insights from affective and cognitive neuroscience to fully understand the social brain of language learning.

## References

[1] Meltzoff A, Kuhl P, Movellan J, Sejnowski T (2009). Foundations for a new science of learning. Science.

[2] Chomsky, N. *Syntactic structures*. Janua Linguarum 4 (The Hague, Mouton, 1957).

[3] Chomsky, N. *Aspects of the Theory of Syntax* (MIT, Cambridge, MA, 1965).

[4] Redcay E, Schilbach L (2019). Using second-person neuroscience to elucidate the mechanisms of social interaction. Nat. Rev. Neurosci..

[5] Babiloni F, Astolfi L (2014). Social neuroscience and hyperscanning techniques: past, present and future. Neurosci. Biobehav. Rev..

[6] Hickok G, Poeppel D (2007). The cortical organization of speech processing. Nat. Rev. Neurosci..

[7] Hagoort P (2019). The neurobiology of language beyond single-word processing. Science.

[8] Ferstl EC (2010). Neuroimaging of text comprehension: Where are we now?. Ital. j. linguist..

[9] Verga L, Kotz SA (2013). How relevant is social interaction in second language learning?. Front. Hum. Neurosci..

[10] Tomasello M (2000). The social-pragmatic theory of word learning. Pragmatics.

[11] Yu C, Ballard D (2007). A unified model of early word learning: Integrating statistical and social cues. Neurocomputing.

[12] Li, P., & Zhao, X. In *Research Methods in Psycholinguistics and the Neurobiology of Language: A Practical Guide* (eds. de Groot, A. & Hagoort, P.) 208–229 (Malden, MA: John Wiley & Sons, Inc., 2017).

[13] Kuhl P, Tsao FM, Liu HM (2003). Foreign-language experience in infancy: effects of short-term exposure and social interaction on phonetic learning. PNAS.

[14] Verga L, Kotz SA (2017). Help me if I can’t: Social interaction effects in adult contextual word learning. Cognition.

[15] Lenneberg EH (1967). Biological Foundations of Language.

[16] Bates, E., & MacWhinney, B. In *Mechanisms of Language Acquisition* (ed. MacWhinney, B.) 157–194 (Lawrence Erlbaum Associates, 1987).

[17] MacWhinney, B. *The Routledge Handbook of Second Language Acquisition* (eds. Gass, S. & Mackey, A.) 211–227 (New York: Routledge, 2012).

[18] Li, P., & MacWhinney, B. In *The Encyclopedia of Applied Linguistics* (ed. Chapelle, C. A.) 1–5 (John Wiley & Sons, Inc., Malden, MA, 2013).

[19] Hernandez A, Li P, MacWhinney B (2005). The emergence of competing modules in bilingualism. Trends Cogn. Sci..

[20] Hernandez A, Li P (2007). Age of acquisition: its neural and computational mechanisms. Psychol. Bull..

[21] Johnson JS, Newport EL (1989). Critical period effects in second language learning: the influence of maturational state on the acquisition of English as a second language. Cogn. Psychol..

[22] Zhao X, Li P (2010). Bilingual lexical interactions in an unsupervised neural network model. IJB.

[23] Kroll JF, Stewart E (1994). Category interference in translation and picture naming: evidence for asymmetric connections between bilingual memory representations. J. Mem. Lang..

[24] Linck J, Kroll J, Sunderman G (2009). Losing access to the native language while immersed in a second language: Evidence for the role of inhibition in second-language learning. Psychol. Sci..

[25] DeLuca V, Rothman J, Bialystok E, Pliatsikas C (2019). Redefining bilingualism as a spectrum of experiences that differentially affects brain structure and function. PNAS.

[26] Grosjean, F. In *The Psycholinguistics of Bilingualism* (eds. Grosjean, F. & Li, P.) 5–25 (Wiley & Sons, Inc., Malden, MA, 2013).

[27] Li, P. In *The Handbook of Language Emergence* (eds. MacWhinney, B., & O’Grady, W.) 511–536 (John Wiley & Sons, Inc., Maiden, MA, 2015).

[28] Lantolf J (2006). Sociocultural theory and L2: State of the art. Stud. Second Lang. Acquis..

[29] Tomasello M (2003). Constructing a Language: A Usage-based Theory of Language Acquisition.

[30] Hall JK (2019). The contributions of conversation analysis and interactional linguistics to a usagebased understanding of language: expanding the transdisciplinary framework. Mod. Lang. J..

[31] Ellis NC (2019). Essentials of a theory of language cognition. Mod. Lang. J..

[32] Tulving E, Thomson DM (1973). Encoding specificity and retrieval processes in episodic memory. Psychol. Rev..

[33] Godden G, Baddeley A (1975). Context-dependent memory in two natural environments: on land and underwater. Br. J. Psychol..

[34] Craik FI, Lockhart RS (1972). Levels of processing: a framework for memory research. J. Verbal Learning Verbal Behav..

[35] Paivio A (1990). Mental Representations: A Dual Coding Approach.

[36] Mayer RE, Moreno R, Boire M, Vagge S (1999). Maximizing constructivist learning from multimedia communications by minimizing cognitive load. J. Educ. Psychol..

[37] Liu C, Wang R, Li L, Ding G, Yang J, Li P (2020). Effects of encoding modes on memory of naturalistic events. J. Neurolinguist..

[38] Marian, V. & Kaushanskaya, M. in *Relations Between Language and Memory*: *Sabest Saarbrucker Beitrage zur Sprach- und Transl* (ed. Zelinsky-Wibbelt, C.) 95–120 (Frankfrut, Peter Lang, 2011).

[39] Marian V, Neisser U (2000). Language-dependent recall of autobiographical memories. J. Exp. Psychol. Gen..

[40] Marian V, Kaushanskaya M (2007). Language context guides memory content. Psychon. Bull. Rev..

[41] Barsalou, L. W., Niedenthal, P. M., Barbey, A. K. & Ruppert, J. A. In *The Psychology of Learning and Motivation: Advances in Research and Theory* (ed. Ross, B. H.) 43–92 (Elsevier Science, 2003).

[42] Glenberg AM, Sato M, Cattaneo L (2008). Use-induced motor plasticity affects the processing of abstract and concrete language. Curr. Biol..

[43] Chomsky N (1981). Lectures on Government and Binding.

[44] Fodor JA (1983). The Modularity of Mind.

[45] Barsalou, L. W. In *Embodied Grounding: Social, Cognitive, Affective, and Neuroscientific Approaches* (eds. Semin, G. R. & Smith, E. R.) 9–42 (Cambridge University Press, 2008).

[46] Aziz-Zadeh L, Damasio A (2008). Embodied semantics for actions: findings from functional brain imaging. J. Physiol. Paris.

[47] Willems RM, Casasanto D (2011). Flexibility in embodied language understanding. Front. Psychol..

[48] Legault J (2019). Immersive virtual reality as an effective tool for second language vocabulary learning. Languages.

[49] Mayer KM, Yildiz IB, Macedonia M, von Kriegstein K (2015). Visual and motor cortices differentially support the translation of foreign language words. Curr. Biol..

[50] Li P, Legault J, Litcofsky KA (2014). Neuroplasticity as a function of second language learning: anatomical changes in the human brain. Cortex.

[51] Grant AM, Fang S-YY, Li P (2015). Second language lexical development and cognitive control: a longitudinal fMRI study. Brain Lang..

[52] Qi Z, Han M, Garel K, Chen E, Gabrieli J (2015). White-matter structure in the right hemisphere predicts Mandarin Chinese learning success. J. Neurolinguist..

[53] Yang J, Gates K, Molenaar P, Li P (2015). Neural changes underlying successful second language word learning: An fMRI study. J. Neurolinguist..

[54] Breitenstein C (2005). Hippocampus activity differentiates good from poor learners of a novel lexicon. NeuroImage.

[55] Tagarelli K, Shattuck K, Turkeltaub P, Ullman M (2019). Language learning in the adult brain: a neuroanatomical meta-analysis of lexical and grammatical learning. NeuroImage.

[56] Jeong H (2010). Learning second language vocabulary: neural dissociation of situation-based learning and text-based learning. NeuroImage.

[57] Legault J, Fang S, Lan Y, Li P (2019). Structural brain changes as a function of second language vocabulary training: Effects of learning context. Brain Cogn..

[58] Stein M, Winkler C, Kaiser A, Dierks T (2014). Structural brain changes related to bilingualism: does immersion make a difference?. Front. Psychol..

[59] Verga L, Kotz SA (2019). Spatial attention underpins social word learning in the right fronto-parietal network. NeuroImage.

[60] Thompson-Schill S (2003). Neuroimaging studies of semantic memory: inferring “how” from “where”. Neuropsychologia.

[61] Bressler S, Menon V (2010). Large-scale brain networks in cognition: emerging methods and principles. Trends Cogn. Sci..

[62] Bassett D, Sporns O (2017). Network neuroscience. Nat. Neurosci..

[63] Li P, Grant A (2016). Second language learning success revealed by brain networks. Biling. Lang. Cogn..

[64] Hagoort P (2005). On Broca, brain, and binding: a new framework. Trends Cogn. Sci..

[65] Abutalebi J, Green D (2007). Bilingual language production: the neurocognition of language representation and control. J. Neurolinguist..

[66] Nichols ES, Joanisse MF (2016). Functional activity and white matter microstructure reveal the independent effects of age of acquisition and proficiency on second-language learning. NeuroImage.

[67] Sun X, Li L, Ding G, Wang R, Li P (2019). Effects of language proficiency on cognitive control: Evidence from resting-state functional connectivity. Neuropsychologia.

[68] Noordzij ML (2009). Brain mechanisms underlying human communication. Front. Hum. Neurosci..

[69] Redcay E (2010). Live face-to-face interaction during fMRI: a new tool for social cognitive neuroscience. NeuroImage.

[70] Hagoort P, Indefrey P (2014). The neurobiology of language beyond single words. Annu. Rev. Neurosci..

[71] Frith C, Frith U (2007). Social cognition in humans. Curr. Biol..

[72] Adolphs R (2009). The social brain: neural basis of social knowledge. Annu. Rev. Psychol..

[73] Ferstl EC, Neumann J, Bogler C, von Cramon DY (2008). The extended language network: a meta-analysis of neuroimaging studies on text comprehension. Hum. Brain. Mapp..

[74] Mason R, Just M (2009). The role of the theory-of-mind cortical network in the comprehension of narratives. Lang. Linguist. Compass.

[75] Li P, Clariana RB (2019). Reading comprehension in L1 and L2: An integrative approach. J. Neurolinguist..

[76] Peñaloza, C., Grasemann, U., Dekhtyar, M., Miikkulainen, R. & Kiran, S. BiLex: a computational approach to the effects of age of acquisition and language exposure on bilingual lexical access. *Brain Lang*., in press, 10.1016/j.bandl.2019.104643 (2020). 10.1016/j.bandl.2019.104643 PMC6692118 31247403

[77] Zhao X, Li P (2013). Simulating cross-language priming with a dynamic computational model of the lexicon. Biling.: Lang. Cogn..

[78] Li P (2009). Lexical organization and competition in first and second languages: computational and neural mechanisms. Cogn. Sci..

[79] Zhang, X., Yang, J., Wang, R. & Li, P. A neuroimaging study of semantic representation in first and second languages. *Lang. Cogn. Neurosci*. In press, 10.1080/23273798.2020.1738509 (2020).

[80] Caldwell-Harris CL (2015). Emotionality differences between a native and foreign language: implications for everyday life. Curr. Dir. Psychol. Sci..

[81] Pavlenko A (2012). Affective processing in bilingual speakers: disembodied cognition?. Int. J. Psychol..

[82] Ivaz L, Costa A, Dunabeitia J (2016). The emotional impact of being myself: emotions and foreign-language processing. J. Exp. Psychol. Learn. Mem. Cogn..

[83] Lambon Ralph MA, Jefferies E, Patterson K, Rogers T (2017). The neural and computational bases of semantic cognition. Nat. Rev. Neurosci..

[84] Pulvermüller F (2013). How neurons make meaning: brain mechanisms for embodied and abstract-symbolic semantics. Trends Cogn. Sci..

[85] Myers L, LeWitt R, Gallo R, Maselli N (2017). Baby FaceTime: Can toddlers learn from online video chat?. Dev. Sci..

[86] Lytle S, Garcia-Sierra A, Kuhl P (2018). Two are better than one: Infant language learning from video improves in the presence of peers. PNAS.

[87] Jeong, H., Li, P., Suzuki, W., Sugiura, M. & Kawashima, R. Neural mechanisms of language learning from social contexts, Manuscript under review (2020). 10.1016/j.bandl.2020.104874 33220647

[88] Dede C (2009). Immersive interfaces for engagement and learning. Science.

[89] Peeters D (2019). Virtual reality: a game-changing method for the language sciences. Psychon. Bull. Rev..

[90] Li P, Legault J, Klippel A, Zhao J (2020). Virtual reality for student learning: understanding individual differences. Hum. Behav. Brain.

[91] Lan YJ, Fang SY, Legault J, Li P (2015). Second language acquisition of Mandarin Chinese vocabulary: context of learning effects. Educ. Technol. Res. Dev..

[92] Hsiao IYT, Lan YJ, Kao CL, Li P (2017). Visualization analytics for second language vocabulary learning in virtual worlds. J. Educ. Techno. Soc..

[93] Freund J (2013). Emergence of individuality in genetically identical mice. Science.

[94] Krokos E, Catherine P, Amitabh V (2019). Virtual memory palaces: immersion aids recall. Virtual Real..

[95] Johnson-Glenberg MC, Birchfield DA, Tolentino L, Koziupa T (2014). Collaborative embodied learning in mixed reality motion-capture environments: two science studies. J. Educ. Psychol..

[96] Nature Editorial. (2015). STEM education: to build a scientist. Nature.

[97] Zhang Y (2009). Neural signatures of phonetic learning in adulthood: a magnetoencephalography study. Neuroimage.

[98] Yusa N, Kim J, Koizumi M, Sugiura M, Kawashima R (2017). Social interaction affects neural outcomes of sign language learning as a foreign language in adults. Front. Hum. Neurosci..

[99] Ullman MT (2001). The neuronal basis of lexicon and grammar in first and second language: The declarative/procedural model. Biling. Lang. Cogn..

[100] Ullman MT, Lovelett JT (2018). Implications of the declarative/procedural model for improving second language learning: The role of memory enhancement techniques. Second Lang. Res..

[101] Goldin-Meadow S, Alibali MW (2013). Gesture’s role in speaking, learning, and creating language. Annu. Rev. Psychol..

[102] Ozyurek, A. In *Oxford Handbook of Psycholinguistics* (eds. Rueschemeyer, S.A. & Gaskell, M.G.) 592–607 (Oxford University Press, 2018).

[103] Ripollés P (2014). The role of reward in word learning and its implications for language acquisition. Curr. Biol. CB.

[104] Dörnyei, Z. In *Individual Differences and Instructed Language Learning* (ed. Robinson, P.) 137–158 (John Benjamins, 2002).

[105] MacIntyre PD, Legatto JJ (2011). A dynamic system approach to willingness to communicate: Developing an idiodynamic method to capture rapidly changing affect. Appl. Linguist..

